# “It’s extremely hard but it’s not a burden”: A qualitative study of family caregiving for people living with dementia in Vietnam

**DOI:** 10.1371/journal.pone.0259788

**Published:** 2021-11-29

**Authors:** Huong Nguyen, Trang Nguyen, Duyen Tran, Ladson Hinton

**Affiliations:** 1 School of Nursing, University of Minnesota, Minneapolis, Minnesota, United States of America; 2 University of Social Sciences and Humanities, Vietnam National University, Hanoi, Vietnam; 3 Department of Neurology, University of California, Davis, Sacramento, California, United States of America; 4 Department of Psychiatry and Behavioral Sciences, University of California, Davis, Sacramento, California, United States of America; University of Haifa, ISRAEL

## Abstract

**Background:**

Vietnam is one of the fastest-aging countries in the world with a rising number of people with Alzheimer’s disease and related dementias (ADRD). Families in Vietnam provide most of the care for persons living with dementia, yet our understanding of their experiences and needs is limited. This study examined the family caregiving experience in a semi-rural region outside of central Hanoi from the perspectives of family caregivers and other key informants.

**Materials:**

Semi-structured interviews were conducted with 21 key stakeholders (12 family caregivers and 9 healthcare providers and community leaders). A descriptive, thematic analysis was conducted.

**Results:**

Qualitative data analysis revealed four themes related to the family caregiving experience: 1) perceptions of dementia symptoms as a normal part of aging rather than a disease, 2) caregiving as a moral and expected familial obligation, 3) patterns of caregiving that are heavily influenced by both gender and sibling order, and 4) multiple challenges or hardships, including time constraints, loss of income, increased social isolation, a toll on their perceived physical health, and emotional distress. Caregivers rejected the notion that caregiving was a “burden” and expressed their distress through terms such as frustration, sadness, and exhaustion.

**Conclusions:**

In this low-resource setting, the stress of family caregiving may be amplified by lack of community resources, cultural stigma discouraging outside help-seeking, and economic impact of care provision. The study highlights the vulnerability and predicament of Vietnamese women who often face multiple challenges in their caregiving role as well as the urgent need for the development of community-based programs and supports.

## Introduction

By the year 2050, it is projected that nearly two-thirds of the people living with dementia will reside in low- and middle-income countries (LMIC), and the vast majority are and will continue to be cared for in the home [1, 2]. While the challenges family caregivers of persons with dementia face are well-documented in high-income countries (HIC), we are still in the early stages of understanding the experience of family caregiving in LMIC where public awareness of dementia is often lower, healthcare systems are less developed, and community support programs may be less available [3]. A better understanding of the experience of family caregiving in LMIC can help tailor support interventions and programs to local cultural values and lower resource settings.

This study focused on the experience of caregiving for persons with dementia in Vietnam, a LMIC in Asia whose elderly population is projected to more than double by 2050 [4] and is just beginning to develop community services and supports for persons with dementia and their families [5, 6]. Understanding the role of filial piety and other Vietnamese cultural values such as spirituality and religion in shaping the caregiving experience can help inform the tailoring of caregiver interventions [7, 8]. Spirituality and religion influence aspects of caregiving such as caregivers’ suffering, their motivations for providing care, and their understanding of the nature of dementia [8]. In this population, dementia symptoms are often viewed as a normal part of aging rather than a disease [7, 9, 10]. While there is a growing literature on Vietnamese family dementia caregiving in the United States (U.S.) and other countries [7–9, 11, 12], very little has been published on the day-to-day lived experience of family caregiving in Vietnam, especially in more rural areas where resources and education about dementia are scarce.

To address this gap, this paper describes the meanings of dementia and the day-to-day lived experience of family caregiving in an area just outside of central Hanoi in Northern Vietnam. Soc Son is a semi-rural area with a primary focus on agriculture (e.g., rice farming) but also new economic and industrial zones. The total population of Soc Son is approximately 330,000 people, living in 25 communes (*xã*) and 1 central town (*thị trấn*). Like many other regions in Vietnam, there are few formal supports available to family caregivers of persons with dementia. Vietnamese older adults living with dementia have been traditionally taken care of primarily by their family members [13], and this is reinforced by laws mandating family responsibility for caring for older adults [14].

Our study is based on thematic analysis of 21 in-depth qualitative interviews conducted with key informants, including family caregivers of persons with dementia, national and local healthcare providers, and community leaders. This study was conducted as part of a larger project as the first step in a process to develop and culturally adapt a family caregiving intervention in Vietnam [15, 16].

## Methods

### Design

The primary goal of this qualitative study is descriptive. Our approach is appropriate given the relative lack of prior published studies on family dementia caregiving experience in Vietnam and builds on prior work that outlines this approach to qualitative research [17–19]. The study was reported using the Standards for Reporting Qualitative Research (SRQR) criteria for reporting qualitative research [20].

### Setting

The study was conducted in Soc Son District (*huyện Sóc Sơn*) located on the outskirts of Hanoi city limits. Soc Son was selected as the study site because of the health system’s strong history of collaboration with the Vietnam National Geriatric Hospital (NGH) and the Ministry of Health, including testing of novel programs and interventions to support older adults. Like many other rural and semi-rural areas outside of the major urban centers, many people with dementia go undiagnosed and there are very few formal supports available to families in the community.

### Research team

This qualitative study was conducted as part of a larger project that is a collaboration between researchers in Vietnam (Vietnam National Geriatric Hospital) and the U.S. For this study, the team was interdisciplinary, including social work (HN and TN), geriatric psychiatry (LH), and psychology (DT). Except one team member (LH), all team members were born in Vietnam and are fluent in Vietnamese. Interviews and primary data analyses were conducted by team members who were fluent in Vietnamese. The project was also conducted in collaboration with Vietnam’s Ministry of Health and the Department of Health at Soc Son District.

### Sample

With the goal of engaging a broad set of perspectives on dementia family caregiving, we conducted purposive sampling with an initial goal of recruiting 24 participants, including 12 family caregivers and 12 non-family key informants, including local and national healthcare providers (e.g., doctors and nurses), and community leaders. However, after 21 interviews (12 family caregivers and 9 non-family key informants), the research team decided that data saturation had been reached and stopped recruitment. Family caregivers who were 18 years old and above with the primary responsibility of caring for a family member with dementia were identified by staff at the local health stations and referred to the study. The local health staff had intimate knowledge about each family in their community. A total of 12 family caregivers, equally male and female, aged from 48 to 62 (average age of 56) participated in the study. Caregivers had a variety of roles, five of the male caregivers were given primary responsibility for decision making and secondary responsibility for providing day-to-day care. The remaining caregivers (1 male and 6 female) provided the most hands-on care in the household. Males were primarily eldest sons taking care of their parents; females were primarily eldest daughters-in-law taking care of their parents-in-law. About 60% of caregivers were rice farmers; a few of them were teachers and construction workers. All participants were living in the same house with care recipients at the time of the interview. Care recipients in this study aged 88 on average (ranging from 83 to 94), with an equal gender distribution between males and females. The severity of their dementia also varied, with some being bed-ridden for years, some manifesting severe cognitive and behavioral problems, and some only starting to show dementia symptoms. Table 1 describes the sample characteristics of caregivers and care recipients.

**Table 1:** Family caregiver and care recipient characteristics (N = 12).

Caregiver				Care Recipient	
ID	Gender	Occupation	Relationship with care recipient	Gender	Dementia severity
CG1	Male	Retired	Son	Female	Severe
CG2	Female	Farmer	Daughter-in-law	Male	Severe
CG3	Male	Retired	Son	Male	Mild
CG4	Female	Farmer	Daughter-in-law	Female	Moderate
CG5	Male	Farmer	Son	Female	Severe
CG6	Female	Farmer	Daughter-in-law	Male	Severe
CG7	Female	Farmer	Wife	Male	Severe
CG8	Male	Farmer	Son	Male	Moderate
CG9	Male	Teacher	Son	Female	Moderate
CG10	Male	Constructor	Son	Male	Mild
CG11	Female	Housewife	Daughter-in-law	Female	Severe
CG12	Female	Farmer	Daughter-in-law	Female	Severe

In addition to family caregivers, nine key stakeholders, including four males and five females, were interviewed (see Table 2). Three were leaders of local organizations such as People’s Committee, Women’s Association, and Association of Older Adults; the rest were staff at local clinics, the district healthcare department, and healthcare providers at the NGH.

**Table 2:** Community leader and healthcare provider characteristics (N = 9).

Stakeholder	Gender	Job/Position	Affiliation
*National level*			
HP4	Female	Healthcare professional	NGH
HP5	Female	Healthcare professional	NGH
HP6	Male	Healthcare professional	NGH
*District level*			
HP1	Male	Healthcare professional	Healthcare Center
*Commune level*			
CL1	Male	Officer	People’s Committee
CL2	Male	Officer	Association of Older Adults
CL3	Female	Officer	Women’s Association
HP2	Female	Healthcare professional	Healthcare Center
HP3	Female	Healthcare professional	Healthcare Center

### Interview

The interview guide had two parts, the first broadly focused on the experience of family caregiving and the second part focused on perceptions of the proposed intervention. Data from the first part of the interview is the focus of this analysis and included a set of open-ended questions to elicit perspectives on the following topics: 1) perceptions of dementia, 2) the day-to-day experiences of family caregiving, 3) community supports and programs for family caregivers, and 4) unmet needs of family caregivers. Interview guides were similar for both family caregivers and non-family informants with some tailoring of questions for each group. This included questions on systems of caring for persons with dementia for non-family informants, and additional questions on lived experience and day-to-day care provision for family caregivers (see Supporting Information for copy of interview guide in English and Vietnamese).

The interview guide was jointly developed by the authors with input from the larger research team, including senior investigators at the Vietnam National Geriatric Hospital. All interviews were conducted in Vietnamese by the first author or other members of the research team within a three-month period from September to December 2016. Interviews were conducted at caregivers’ homes, key stakeholders’ offices, or in another setting if preferred by participants. The interviews lasted approximately 45 minutes to an hour and were audiotaped.

### Analysis

All interviews were transcribed, cleaned, and de-identified. A separate Excel sheet was created to link transcripts to participants’ demographic characteristics. All identifiable electronic data were maintained on an encrypted device requiring a password for access. Only research team members had access to the database. Two members of the research team, who were bi-lingual in Vietnamese and English, performed data analysis, using thematic analysis [21]. The first author, who conducted most of the interviews, and her graduate research assistant conducted the analyses using multiple steps. First, they read through the transcripts to familiarize themselves with the data, conducted open coding to identify emerging themes using the qualitative analysis software MAXQDA 12, and developed an initial codebook. They then compared the codes, discussed, and revised the codebook. The first author then supervised her graduate research assistant to complete a second round of coding of all interviews. They met weekly to discuss the coding and refine the themes.

### Ethical approval

The Institutional Review Boards (IRB) of the University of California, Davis (UCD), the University of South Carolina (USC), and the Vietnam National Geriatric Hospital (NGH) approved the study protocol. Informed consent was obtained before interviews.

## Results

Each of the following four key themes related to family caregiving experience emerged and were supported by data from interviews with both family caregivers and non-family informants: 1) perceptions of dementia symptoms as a normal part of aging rather than a disease, 2) family caregiving as moral obligation, 3) common family arrangement towards caregiving, and 4) multiple challenges or difficulties, including time constraints, loss of income, increased social isolation, worsened physical health, and emotional distress.

### *“They think it’s natural”*: Perceptions of dementia

Nearly all participants, including both family caregivers and key stakeholders, endorsed the view that most people in the community view dementia as a normal part of aging. The most frequent term used locally to refer to people with dementia was “*lẫn*,” which roughly translated to being “confused,” “forgetful,” “mistaken,” or “lost.” The term “*lẫn”* was used to refer to varying levels of forgetfulness, from mild occasional forgetting to much more severe memory loss and confusion. Caregivers were likely to refer to the older adults with more severe forgetfulness as having “forgetting illness/confusion disease” (*bệnh lẫn*) or “an illness of old age” (*bệnh tuổi già*). Even more extreme forms of memory loss were understood as a normal part of aging, particularly among the very old.

None of the families we interviewed were familiar with the professional term “dementia” (*sa sút trí tuệ*). Healthcare providers and other local leaders said the awareness of dementia in the community was quite low in Soc Son as well as in other parts of Vietnam:

*Many old people in Soc Son are forgetful*. *It is not a small number*. *But people do not know so they do not call it “dementia” [sa sút trí tuệ]*, *they usually call it* “*being forgetful” [lẫn or bệnh lẫn]*. *And not just here but it is like that everywhere*. *(Male healthcare professional at local hospital*, *HP1)*

Staff at the clinic referred to above had only recently become aware of dementia as a medical condition through a public health training program conducted by physicians from the NGH. One consequence of the normalization of dementia symptoms is that families rarely sought help from the healthcare system unless their family member displayed severe emotional and behavioral problems. Several caregivers shared stories about their relatives being diagnosed with dementia only after older adults came to commune health stations for public health programs to check common illnesses such as high blood pressure.

*Here*, *they [commune health stations] often announced about free vision checkup*, *health checkup*, *or other public health programs*, *so he [the care recipient] went to check by himself*. *He went all the time*. *And they said he had a neurological problem*. *(Female caregiver*, *CG7)*

### “*How life happens*”: Family caregiving as moral obligation

In most families, persons with dementia lived in a multigenerational household. Caregiving was an expected obligation and a matter of honor, pride, as well as an emotional bond between parents and children. As both family caregivers and non-family informants told us, caregiving was viewed as something that families “*must do*” in order to serve parents (*phụng dưỡng*) and “*payback*” (*báo hiếu*). As one caregiver stated: “*Children must take care of parents in old age*, *that’s just the law of life*” *(Male caregiver*, *CG1)*. Another caregiver told us: “*We work hard to take care of our parents*, *then our children will do the same to us*. *It comes around like that*” *(Female caregiver*, *CG2)*. The moral obligation to care for parents reflects Confucian precepts of filial piety (*đạo hiếu*), a deeply held set of values dictating that younger generations must respect and care for older ones. The moral obligation of family caregiving was also reinforced by Buddhism through the notion that taking care of family members, especially parents, earned a person good karma and merits, thus helping him to flourish in the present and next lives.

Families “*accepted*” caregiving for their parents with dementia as a normal part of life, much like they accepted their parents’ dementia as a normal part of aging. Because of this normalization of caregiving for parents, “*just do it*” was the overarching motto of caregivers as a healthcare provider observed:

*Now*, *caregivers do not really have a clear idea about taking care of a forgetful person*, *a person with dementia*, *and a normal old person*. *When they must take care of someone or something*, *they just do it*. *(Male healthcare professional at local hospital*, *HP1)*

While many family caregivers we interviewed faced significant challenges in providing care to people living with dementia, there was a sense of resignation and acceptance of caregiving as something that “*must be done*.” There was also a reluctance to seek help because caregiving was viewed as a family responsibility and reaching out to others outside the family signified a moral failure on the part of the family and risked “*loss of face*” in the eyes of the broader community. Additionally, from participants’ views, formal resources to assist caregivers were unavailable, limiting options for seeking outside help.

### “*Being a daughter-in-law means you must take care of your parents-in-law*”: Gender and birth order in family caregiving

As described by both family caregivers and non-family informants, caregiving patterns were heavily influenced by both gender and birth order, with primary responsibility for the day-to-day care of persons with dementia being shouldered by the eldest son’s wife. This pattern is consistent with Vietnamese culture, in which it is traditional for the parents to live with and be cared for by the eldest son (*trai trưởng*) and his family, with other siblings and their families playing a supportive role. In our study, 75% (9/12) of the older adults with dementia were living with the eldest son, his wife, and even grandchildren.

While residing with the eldest son was the most common pattern observed in the family caregivers we interviewed, there were exceptions. In one situation, the person with dementia’s eldest son passed away, so he lived with his daughter-in-law. In another case, the care recipient lived with the youngest son because both parties and the whole family agreed that the youngest son was “*the most suited*” (“*hợp nhất”*, *i*.*e*., *emotionally closest*) to care for his mother based on the closeness of their relationship. In yet another case, a wife was the primary caregiver to her husband. She explained to us: “*Our children*, *they still have to work to raise their own children*. *We cannot leave it [the caregiving] to them” (Female caregiver*, *CG7)*. In almost all cases, however, there was a clearly designated family member providing most of the hands-on care to the person with dementia, with other family members expected to contribute time, money, and efforts to various degrees depending on availability, negotiation, and family dynamics.

Within households, the caregiving roles of the eldest son and his wife were usually highly differentiated. The eldest son was recognized by their family and local community as the official person “in charge” of the overall caregiving responsibilities. In this role, they were involved in coordinating with other family members and making major decisions related to elder care, such as major medical decisions. As we learned, the eldest son also played an important gatekeeper role in decisions about research participation. Most of the hands-on caregiving was done by the eldest daughter-in-law, who would cook, feed, bathe, clean, wash, walk, sit with, talk to, and stay all the time with the parent with dementia. One daughter-in-law described the standard care arrangements:

*Often the parents live with the eldest son while the eldest daughter-in-law will do the caregiving*. *If other children live nearby*, *they can lend a hand here and there*, *adding a little bit of help*. *It is like that everywhere*, *not just here*. *But those caring tasks like feeding*, *bathing*, *washing laundry are women’s job*. *(Female caregiver*, *CG2)*

While men were usually not the ones providing most of the hands-on caregiving, they often helped with certain “heavy duty” tasks (such as carrying the bed-ridden parent from bed to the bathroom for bathing). Other family members provided varying levels of support to the primary caregiver. Support might come in the form of cash, food, visiting the person with dementia, showing emotional support, and occasionally helping with hands-on care. The caregiver’s children might also provide help, depending on how emotional and spatially close to the grandparents they felt, or whether their parents ordered them to help. However, support from other family members could not always be relied upon:

*I must do everything alone from A to Z*. *All the hard work*, *from start to end*. *You see*, *it is very hard being the eldest child; you must take care of your parents*, *you have your own work*, *and you have this task or the other task to complete too*. *Who else will do it for you*? *The brothers and sisters only come by every now and then*. *(Female caregiver*, *CG4)*

### “*They just suffer alone*”: Difficulties and challenges of family caregiving

In families, daughters-in-law often provided the most time-intensive, hands-on, day-to-day care. In the interviews, both family caregivers and non-family informants described the difficulties of caregiving, including both the time-intensive and often physically demanding nature of day-to-day care, as illustrated in the quotation above, as well as taking a social, financial, and emotional toll on caregivers.

#### Time demands

“Having no time” was the most common complaint among caregivers in this study, especially among the female hands-on caregivers. They described their caregiving routines as “around the clock,” from early morning until late at night and even throughout the night when the care recipient might need to get up to go to the toilet or talk too loudly, preventing them from sleeping. Daily caregiving tasks included cooking, feeding, bathing, managing medications, tending to all other needs of care recipients, and keeping an eye on care recipients’ safety. A widowed daughter-in-law taking care of her parents-in-law sobbed uncontrollably as she described her overwhelming daily routines:

*Early morning*, *I start taking care of them; I must cook for them*, *then give them medicines 3 times per day as the doctor said so*, *then giving them a bath*, *cleaning them*. *I have no time to rest*. *And then I also raise some chicken to have a bit of income*. *You see*, *it is very hard*, *my life is too difficult*. *Sometimes I just want a little of time for myself*, *but there is none*. *(Female caregiver*, *CG2)*

Another caregiver echoed the “no time to rest” theme, noting that she did not even have time to rest at noon time, as is typical of most Vietnamese living in rural areas to avoid the midday heat, because she had to keep an eye on the mother-in-law with dementia:

*It [caregiving] is around the clock*, *I have no time for rest at noon*, *I take my grandchild home from school at noon*, *give him lunch*, *then let him nap a little bit*, *then take him back to school*, *then go home to take her [care recipient] to the toilet*. *She [urinates and defecates] multiple times per day*, *it is not like it is just one time*. *(Female caregiver*, *CG4)*

Healthcare providers and community leaders had observed that some caregivers seemed “depressed,” “exhausted,” or “hopeless.” A healthcare provider recalled witnessing caregivers purposefully leaving their parents at the clinic so that they could take a break to rest:

*There are cases where they [caregivers] brought their parents here [to the clinic] and they felt very depressed because they did not see a way for their parents to become normal again*. *They spent so much time providing care that they felt exhausted and hopeless*. *(Female healthcare professional at national hospital*, *HP5)*

#### Loss of income

One consequence of the time-intensive nature of caregiving was loss of income. This constituted a major source of worry for caregivers in this study. Providing “around the clock” care meant giving up or severely curtailing the amount of time working outside the home. One caregiver told us about her need to give up farm work:

*Like farming*, *I had to give it up; I only do a little bit of that; just to make enough rice for three people*. *Before*, *I worked on the rice field and raised pigs and chickens*. *Now I must give up raising poultry*. *I only have a few chickens*. *My income went down a lot*. *Before*, *I worked a lot in the farm*, *I sold the extra for cash and have some savings*. *Now I can only make enough for my family’s meals*. *(Female caregiver*, *CG2)*

Even male caregivers who were not providing hands-on caregiving expressed a strong sense of being limited in their work and mobility because they had to factor in their sick parents when making decisions about jobs. They could not accept jobs far from home, and they did not even go far from home to visit friends for fear that they would not be able to come home fast enough to take care of their sick parents. An eldest son explained his thoughts:

*Honestly*, *we are the eldest children*, *we cannot go far [for jobs] anymore*. *Our parents are old now*, *we cannot go far*. *Even when other men told me to come over their house for a drink*, *to be honest to you*, *I could not go anymore*. *I only work around the village in case something happens*, *then my children call*, *and I can get home right away*. *(Male caregiver*, *CG8)*

Healthcare providers and community workers observed that many caregivers were put into the situation of having to choose between outside work to meet financial needs or spending time caring for their parents. If they decided to maintain their jobs, they would have to spend money hiring a housemaid or helper, an option that many families could not afford. A healthcare provider emphasized financial hardship as a critical issue for families:

*Really*, *for families with someone with Alzheimer’s disease*, *especially at the last stage*, *it is truly a disaster for the family because they must hire professional caregivers or hire someone to watch over the patient*, *then the children must quit jobs*, *then even the grandchildren must take shifts with caregiving*. *It’s a great burden to the family members*, *perhaps not emotionally*, *but financially*, *it’s a great burden*. *(Male healthcare professional at national hospital*, *HP6)*

Several changes over the past several decades have contributed to the difficult financial predicament that many families face when caring for persons with dementia. Family size has shrunk in recent years due to the implementation of family planning policies, so there are fewer in the younger generation to support the elder. In addition, with the development of new economic and industrial zones, many younger people now work in factories in new urban areas with less flexible schedules compared to farm work. Families who had neither the resources to hire outside help nor the ability to survive on one income faced extremely difficult choices. One healthcare provider said they were aware of other families who had resorted to locking the parents with dementia at home during the day when they had to go to work:

*It is the era of market economy now*. *Children must work*, *they cannot stay at home with their parents all day*, *so they lock the door when go to work*. *Otherwise*, *their parents may wander out of the house and get lost or fall into a pond*. *So*, *sons or daughters must lock the door or gate when they go to work*. *Maltreatment is rare here*. *Most of the adult children love their parents*. *(Male local community leader*, *CL1)*

#### Worsening of physical health

While the adverse impacts, particularly economic, were felt by male and female caregivers alike, the physical, social, and emotional toll of caregiving was felt most acutely by daughters-in-law because they provided the most time-intensive and demanding aspects of caregiving. More than half of the caregivers expressed various concerns about the decline of their health due to caregiving such as lack of proper eating schedule, poor appetite, and lack of quality sleep. The mere amount of caregiving tasks were common factors contributing to their concerns about overall health:

*I lost a lot of weight*. *I used to be chubby and white*, *but I lost a lot of weight this year*. *My health declined a lot*. *I had gone to the doctor*, *I had stomachache and loss of sleep*, *so my blood pressure went up; every month I had to go get medicines*. *My head was always fuzzy and clouded*. *(Female caregiver*, *CG2)*

In providing care, sometimes caregivers had to use physical strength to carry their parents from bed to bathroom, to wheelchair, or to the front yard, all of which negatively affected their health. The physical challenges of caregiving were described by a daughter-in-law:

*I take care of bathing and washing laundry for him*. *He cannot do it himself*. *I carry him to the bath myself*. *My husband and children go to work until 9 or 10pm*. *My father-in-law is 40 kgs*. *I am 46 kgs*. *It is difficult to carry him because his body is stiff*, *it is not flexible*. *I am always scared of slipping or falling*. *Just a short distance to the bed but I am scared*. *(Female caregiver*, *CG4)*

A healthcare provider observed that *“caregivers sometimes were so exhausted at night that they fainted*, *they passed out*, *and the nurses had to take care of them” (Female healthcare professional at national hospital*, *HP5)*.

#### Increased social isolation

There was also a loss of social life, especially among female caregivers. Caregiving demands left little time to spend with neighbors or friends. They often could not attend community events or family events, such as weddings, funerals, and celebrations:

*I must select; if a wedding is on a Sunday and my husband is at home*, *then I can go*. *Or if he is not at home but I can ask one of his sisters to come*, *then I can go*. *If his sisters are busy*, *then I am stuck*, *I cannot do anything about it*. *(Female caregiver*, *CG4)*

Some had to give up their past membership or leadership roles in the community’s various social organizations while others had to be very selective about what social role they could still maintain. A female caregiver described her situation:

*[Caregiving] affects me a lot*. *All my social activities before*, *I had to give up*. *Like before*, *I participated in the women’s union*, *I was in the chairing committee of the union; but since my father became like this*, *I had to give it up*, *I could not go anymore*. *(Female caregiver*, *CG7)*

Other caregivers felt resentment that others in the family did not do enough to support them resulting in tension and contributing to their sense of isolation. A widowed caregiver who was taking care of her parents-in-law told us:

*I do not mind the workload*, *but I feel so pity for myself*. *Other women*, *when they get married*, *they can rely on their husbands*. *But me*, *I am alone*, *my husband left me with his old parents and two young children*. *My head is always so heavy and dizzy*. *Often*, *I felt sad*. *Sometimes I thought to myself I would rather die*, *why my life had so much suffering*. *(Female caregiver*, *CG2)*

Caregivers’ social isolation was amplified by a lack of local community organizations providing supports or service to persons with dementia and their families. While many local organizations exist, such as the local women’s union, people’s committee, and senior clubs, these organizations have yet to develop programs geared towards the needs of caregivers and persons with dementia.

#### Emotional distress

The emotional toll on caregivers was quite apparent in many of the interviews. While telling us their stories, several of the female caregivers broke out in tears:

*Sometimes I didn’t want to live anymore*. *I cried a lot at home*. *My children knew*. *The other day*, *it rained*, *and I stayed home*, *so I opened my daughter’s diary*. *She wrote in the diary “My poor mother*!*” So*, *tears just poured out from me*. *I did not want her to worry*. *(Female caregiver*, *CG2)*

Caregivers used many other words that suggested hardship, challenges, difficulties, frustration, worry, pity for oneself, and an array of complex emotions associated with caregiving. The most frequent word used by caregivers was “*vất vả*,” a word indicating that someone had to use a lot of time, physical strength, and efforts on something that is very difficult. The term also conveys being occupied, busy, and consumed all the time with that task, resulting in emotional stress and exhaustion. They used other words to express their emotions when carrying caregiving tasks such as “sad, depressed” (*buồn*, *chán*), “so shameful, pitiful” for oneself (*nhục lắm*), “suffering/miserable” (*khổ*), and “enslaving/serving” (*hầu hạ*).

Distress was voiced most clearly by women, many of whom were caregivers to their parents-in-law. In some cases, there was added distress because of the perception that the husband or others in the family were not doing their fair share. A healthcare provider observed that “*there are many cases where daughters-in-law had the most pressure and they showed signs of being depressed; I just started to ask [about caregiving] and they burst out crying” (Female healthcare professional at national hospital*, *HP5)*. In many of the interviews we conducted, women cried as they told their stories.

While caregivers often felt distressed, staff at local clinics and community leaders explained that family caregivers had great pressure to keep up with the image of “a good child taking care of his sick parent,” thus they would never complain publicly about caregiving. A healthcare provider mentioned that *“of course*, *it [caregiving] is tiring*. *Very tiring*. *They [caregivers] do complain about that*, *but they dare not complain publicly” (Female healthcare professional at local hospital*, *HP2)*. Another healthcare provider observed that caregivers often suffered in silence because “complaining” would reflect negatively on the family:

*They kept it to themselves; sometimes they could tell their daughters-in-law if the two got along well; if they did not get along well*, *then they could not talk*, *could not share anything; they could not say*, *“Oh I felt this hard*, *I felt that much misery*.*” Sometimes they had to go over the neighbors to share but if their husbands heard*, *then their husbands would say “oh*, *you go and talk bad about your husband’s family*, *about your mother-in-law*.*” (Male healthcare professional at local hospital*, *HP1)*

Despite their distress, caregivers were reluctant to describe caregiving as a “burden” (*gánh nặng*). Healthcare providers and community leaders explained that caregivers avoided the word “burden” because it connoted complaining or being resentful and was in direct conflict with the moral obligation of caregiving based on filial piety. The term “burden,” when translated literally into Vietnamese as “*gánh nặng*,” evokes the image of someone being forced to carry a heavy weight on one’s back, making him miserable yet he might not be able to put it down even if one wished to. To be seen by neighbors or others (including researchers) in the community as complaining risked a loss of face for not only the caregiver but also the entire family.

## Discussion

To our knowledge, this is one of the first studies to examine the experience of family dementia caregiving in Vietnam, and one of relatively few conducted in LMIC. Our study provides a window on the family dementia caregiving patterns and challenges in a semi-rural area in Northern Vietnam where community support programs for families caring for people living with dementia are almost non-existent. Within households, gender heavily influenced patterns of caregiving, with women (often daughters-in-law) taking on primary responsibility for day-to-day “hands-on” care and supervision, with variable support from other family members and little formal community support, and men playing a prominent role in decision-making. Family caregivers experience multiple challenges, including time constraints, negative financial impact, social isolation, and a perceived toll on their physical health and emotional well-being.

Our study highlights the vulnerability and predicament of Vietnamese women who often face many challenges in their caregiving role. While men and other family members often helped with various aspects of caregiving, most of the time-intensive aspects of caregiving were the responsibility of women. Our findings are consistent with a recent report on dementia caregiving in LMIC which concluded that 81% of the hours of caregiving are provided by women [2] and a global survey of caregiving by the 10/66 group highlighting the disproportionate impact of caregiving on women in many countries [22]. In our interviews, women were trying to walk a fine line between adhering to cultural values of filial piety and respect towards older adults, while at the same time trying to give an accurate account of their difficulties, distress, and suffering without appearing to complain about the burden of caregiving. One previous study conducted in Vietnam highlighted a psychological process in which caregivers move through experience, acknowledgement, experiment, and eventual acceptance of the caregiving situation [23]. In our study, caregivers also seemed to have accepted or be resigned to their situation while also emphasizing hardship and difficulties, and even desperation. Both studies, as well as studies of Vietnamese caregivers in the U.S. and Australia [7, 11], highlighted the challenges and emotional distress associated with caregiving for many of our participants.

The theme of social isolation and lack of support outside of the family was prominent in our study. Like many other LMIC, Vietnam lacks community-level programs to support caregivers and has a primary healthcare system that for the most part lacks the capacity for dementia care, including diagnosis and family support [1]. Equally important in contributing to the isolation of families is the lack of public awareness of dementia as a medical problem and the strong cultural expectation of family responsibility for elder care. As several participants told us, seeking help outside the family risked “loss of face” and potential stigmatization. The combination of these factors resulted in families “going it alone” with the greatest impacts often experienced by women. The lack of community-based supports magnifies caregiving stress and highlights an important difference from caregivers in the Vietnamese diaspora who reside in the U.S., Australia, and other HIC where community supports are more available and knowledge of ADRD is higher although stigma, service access, utilization, and cultural appropriateness of interventions continue to be issues for Vietnamese caregivers even in HIC [7–9, 11, 12].

Our study has several practical implications for the adaptation of multi-component caregiver interventions for use in Vietnam. Because male heads of the household (often sons) often have important gatekeeping and decision-making roles in elder care, engaging their support at the beginning of the research process may be important to facilitate recruitment of other family members who may be more involved in day-to-day care. Intervention approaches may also need to allow for the inclusion of multiple family members as many people with dementia live in multi-generational households. Given the low levels of knowledge about dementia and high levels of stress among caregivers, the educational, problem-solving, and stress-reduction components of interventions may be highly relevant. Because of the low level of public awareness of dementia, the educational component may require more time compared with HIC. Finally, many caregivers seemed to experience some relief in simply being able to tell their story. As we reported elsewhere, an important part of training interventionists is to allow the caregiver tell their story as part of the intervention and to listen empathically [24].

This study has several limitations. Our study is based on a relatively small sample of caregivers and stakeholders from one semi-rural area in Vietnam and may not be representative of other parts of Vietnam, particularly areas that are more rural or urban. In addition, the family caregivers in our study were caring for older adults with more advanced dementia and intense caregiving needs and may not reflect the experience of families caring for older adults with milder dementia. Future research is needed to examine the experience of caregiving for older adults with milder dementia as well as those residing in more rural and urban areas. Future studies can also examine the differences in caregiving issues among areas severely affected by the Vietnam War and those not much affected. Moreover, a comparison of perspectives of family caregivers and other key informants was beyond the scope of this analysis but might be explored in future studies.

## Conclusion

Our study contributes to our understanding of the experience and impacts of caregiving for families in Vietnam and adds to research on caregiving experience in lower resource settings [25]. In this low-resource setting, the stress of family caregiving is amplified by lack of community resources, cultural stigma discouraging outside help-seeking, and the adverse economic impact of care provision. The financial impact of elder care deserves special emphasis. Poverty remains a global threat to public health and political stability, particularly in LMIC [26]. Family dementia caregiving is time-intensive, and several participants in our study reported lost income due to cutting back on outside work or giving up jobs completely. Family dementia caregiving in the absence of adequate community supports and other safety nets risks a downward economic trajectory for families that places them at increased risk of falling into poverty. A better understanding of the economic costs of family dementia caregiving is urgently needed to inform policy makers in Vietnam and other LMIC and to build momentum for strengthening supports for family caregivers at the local level, including increasing public awareness, strengthening the capacity of local health systems, and developing community support programs attached to existing groups and programs [27].

## Supporting information

S1 Appendix Interview guide. (DOCX)
