# Supplementary material for: “It’s extremely hard but it’s not a burden”: A qualitative study of family caregiving for people living with dementia in Vietnam
Source: PLoS One. 2021 Nov 29;16(11):e0259788. doi: 10.1371/journal.pone.0259788 (PMC8629297; doi:10.1371/journal.pone.0259788)
Supplement: S1 Appendix — (DOCX) [file pone.0259788.s001.docx]

Qualitative interview guide

1) Perceptions of dementia

1. When you hear the term “dementia,” what do you normally think of?
2. What are some words/terms that you have heard people use to refer to dementia?
3. Is dementia a common problem in this community?
4. Where do you normally see or hear about people with dementia?
5. From whom do you normally see or hear about people with dementia?
6. Do you think people in the community share their thoughts about dementia or they think differently?
7. Do you notice any stigma or discrimination against people with dementia?

2) The day-to-day experiences of family caregiving

- *For caregivers, this section should start with a few questions about the person with dementia that is being taken care of. We can ask caregivers the following questions:*

1. Please describe the relationship between you and the person with dementia, your living arrangement, manifestations of dementia, the daily routines of care.
2. Are multiple people in the family taking care of the person with dementia? How do they divide work? Are tension or conflicts between family members about caretaking?
3. What are the most important challenges family caregivers face in caring for someone with dementia?

- *For healthcare staff and local authorities, this section can start with a few questions about the nature of their work and how it relates to elder care, especially care for people with dementia. Remember to ask them to elaborate instead of just saying “Yes” or “No”. Ask them to provide descriptions.*

1. In your experience, do most people with dementia in your community have someone in the family care for them?
2. In your experience, do you know or hear of cases in which people with dementia do not have any family members to take care of them?
3. In your experience, have you seen, known, or heard of cases in which people with dementia are maltreated by family members such as hitting, insulting, scolding them, belittling them, not feeding them, not giving them necessary medicines, chaining them, locking them, etc.?
4. In your experience, how often do you see cases in which people with dementia are taken care of by multiple family members? In those cases, how do they divide work? Are there conflicts between caregivers and how do they resolve? How do the conflicts affect the care and the person with dementia?
5. In your experience, what challenges do family caregivers face in caring about basic daily activities of someone with dementia? These activities include eating, drinking, bathing, using the toilet, or sleeping.
6. In your experience, what challenges do family caregivers face in caring for the need to moving inside and outside the house of someone with dementia? This need includes visiting neighbors, relatives, attending community social events, going to the hospitals, etc.
7. In your experience, what challenges do family caregivers face in caring about the emotions and moods of someone with dementia? What emotions and moods do you most often see in people with dementia? How does that affect family members?
8. In your experience, what challenges do family caregivers face when dealing with the loss of memory of someone with dementia?
9. In your experience, what challenges do family caregivers face in caring for aggressive or any unpredictable behaviors of someone with dementia?
10. In your experience, what challenges in keeping their daily life, job, and their own family life do family caregivers face in caring for someone with dementia?
11. Based on everything we have discussed, can you name top three challenges that family caregivers face in caring for someone with dementia?

3) Community supports and programs for caregivers

1. Are there community groups, organizations, or services to support caregivers?
2. *For caregivers:* Have you and your family received support from them?
3. What kinds of support do they provide (financial, education, information, emotional support, etc)?
4. Do you think the support is effective and where it’s effective? If it’s not effective, what needs to be done to make it effective?
5. Do you see potential for more support through existing organizations or do you have ideas about new support groups/organizations?

- Local People’s Committee or any other local authorities?
- Local health care system?
- Local education system?
- Local social work centers?
- Local Red Cross?
- Seniors’ club?
- Veterans’ club?
- Farmers’ society?
- Women’s union?
- Youth union?
- Buddhist temples, churches, and other religious/spiritual organizations?
- Self-organized organizations or society such as Seniors’ badminton club or Chess club, etc?

4) Address unmet needs of family caregivers

1. What kinds of help or services are needed to better support family caregivers?

- Health and mental health services?
- Education services?
- Transportation services?
- Housing services?
- Job services?

1. How can healthcare providers better support caregivers?

**Hướng dẫn phỏng vấn định tính**

**1) Nhận thức về chứng sa sút trí tuệ (SSTT)**

1. Khi nghe đến thuật ngữ “sa sút trí tuệ,” anh/chị thường nghĩ đến điều gì?

2. Một số từ/thuật ngữ mà anh/chị đã nghe mọi người sử dụng để nói về chứng SSTT là gì?

3. SSTT có phải là một vấn đề phổ biến trong cộng đồng này không?

4. Anh/chị thường nhìn thấy hoặc nghe nói về những người bệnh SSTT ở đâu?

5. Anh/chị thường nhìn thấy hoặc nghe nói về những người bệnh SSTT từ ai?

6. Anh/chị có nghĩ rằng mọi người trong cộng đồng chia sẻ suy nghĩ của họ về chứng SSTT hay họ nghĩ khác?

7. Anh/chị có nhận thấy bất kỳ sự kỳ thị hoặc phân biệt đối xử nào đối với những người bệnh SSTT không?

**2) Trải nghiệm việc chăm sóc người thân bị bệnh SSTT tại nhà**

*• Đối với người chăm sóc, phần này nên bắt đầu bằng một vài câu hỏi về người bệnh SSTT đang được chăm sóc. Chúng ta có thể hỏi người chăm sóc những câu hỏi sau:*

1. Hãy mô tả mối quan hệ giữa anh/chị và người bệnh SSTT, cuộc sống sinh hoạt của anh/chị, những biểu hiện của bệnh SSTT, những thói quen chăm sóc hàng ngày.

2. Có nhiều người trong gia đình cùng chăm sóc người bệnh SSTT không? Họ phân chia công việc như thế nào? Có căng thẳng hoặc xung đột giữa các thành viên trong gia đình về việc chăm sóc không?

3. Những thách thức quan trọng nhất mà người chăm sóc gia đình đối mặt trong việc chăm sóc người bệnh SSTT là gì?

*• Đối với nhân viên y tế và chính quyền địa phương, phần này có thể bắt đầu bằng một số câu hỏi về bản chất công việc của họ và nó liên quan như thế nào đến việc chăm sóc người cao tuổi, đặc biệt là chăm sóc người bệnh SSTT. Hãy nhớ khuyến khích họ nói rõ hơn thay vì chỉ nói “Có” hoặc “Không.” Yêu cầu họ cung cấp mô tả.*

1. Theo kinh nghiệm của anh/chị, hầu hết những người bệnh SSTT trong cộng đồng của anh/chị có người thân trong gia đình chăm sóc cho họ không?

2. Theo kinh nghiệm của anh/chị, anh/chị có biết hoặc nghe nói về những trường hợp mà người bệnh SSTT không có người nhà chăm sóc họ không?

3. Theo kinh nghiệm của anh/chị, anh/chị đã từng thấy, biết hoặc nghe nói về những trường hợp người bệnh SSTT bị người thân trong gia đình ngược đãi như đánh đập, lăng mạ, mắng mỏ, coi thường họ, không cho ăn, không cho họ uống thuốc cần thiết, xích họ lại, khóa họ lại, v.v.?

4. Theo kinh nghiệm của anh/chị, anh/chị thường thấy những trường hợp người bệnh SSTT được người thân trong gia đình chăm sóc như thế nào? Trong những trường hợp đó, họ phân chia công việc như thế nào? Có xung đột giữa những người chăm sóc không và họ giải quyết như thế nào? Những xung đột ảnh hưởng đến việc chăm sóc và người bệnh SSTT như thế nào?

5. Theo kinh nghiệm của anh/chị, những người chăm sóc gia đình đối mặt với những thách thức nào trong việc quan tâm đến các hoạt động cơ bản hàng ngày của người bệnh SSTT? Những hoạt động này bao gồm ăn, uống, tắm rửa, đi vệ sinh, hoặc ngủ.

6. Theo kinh nghiệm của anh/chị, những thách thức nào mà người chăm sóc gia đình đối mặt với nhu cầu đi lại/di chuyển trong và ngoài nhà của người bệnh SSTT? Nhu cầu này bao gồm thăm hàng xóm, họ hàng, tham dự các sự kiện xã hội cộng đồng, đến bệnh viện, v.v.

7. Theo kinh nghiệm của anh/chị, những người chăm sóc gia đình đối mặt với những thách thức nào trong việc quan tâm đến cảm xúc và tâm trạng của người bệnh SSTT? Anh/chị thường thấy những cảm xúc và tâm trạng nào nhất ở những người bệnh SSTT? Điều đó ảnh hưởng đến các thành viên trong gia đình như thế nào?

8. Theo kinh nghiệm của anh/chị, những người chăm sóc gia đình đối mặt với những thách thức nào khi đối phó với tình trạng mất trí nhớ của người bệnh SSTT?

9. Theo kinh nghiệm của anh/chị, những người chăm sóc gia đình đối mặt với những thách thức nào khi đối phó những hành vi hung hăng hoặc bất kỳ hành vi khó lường nào của người bệnh SSTT?

10. Theo kinh nghiệm của anh/chị, những thách thức nào trong việc duy trì cuộc sống hàng ngày, công việc và cuộc sống gia đình của họ mà những người chăm sóc gia đình đối mặt trong việc chăm sóc người bệnh SSTT?

11. Dựa trên tất cả những gì chúng ta đã thảo luận, anh/chị có thể kể tên ba thách thức lớn nhất mà người chăm sóc gia đình đối mặt trong việc chăm sóc người bệnh SSTT không?

**3) Hỗ trợ cộng đồng và các chương trình dành cho người chăm sóc**

1. Có các nhóm cộng đồng, tổ chức, hoặc dịch vụ để hỗ trợ người chăm sóc không?

2. *Đối với người chăm sóc:* Anh/chị và gia đình anh/chị đã nhận được sự hỗ trợ từ họ chưa?

3. Họ cung cấp những hình thức hỗ trợ nào (tài chính, giáo dục, thông tin, hỗ trợ tinh thần, v.v.)?

4. Anh/chị có nghĩ rằng hỗ trợ đó có hiệu quả không và nó có hiệu quả ở đâu? Nếu nó không hiệu quả, cần phải làm gì để làm cho nó hiệu quả?

5. Anh/chị có thấy tiềm năng nhận được nhiều hỗ trợ hơn thông qua các tổ chức hiện tại hoặc anh/chị có ý tưởng về các nhóm/tổ chức hỗ trợ mới không?

- Ủy ban nhân dân địa phương hoặc bất kỳ chính quyền địa phương nào khác?

- Hệ thống chăm sóc sức khỏe địa phương?

- Hệ thống giáo dục địa phương?

- Các trung tâm công tác xã hội ở địa phương?

- Hội Chữ thập đỏ địa phương?

- Câu lạc bộ

**4) Giải quyết các nhu cầu chưa được đáp ứng của người chăm sóc gia đình**

1. Những loại trợ giúp hoặc dịch vụ nào cần thiết để hỗ trợ người chăm sóc gia đình tốt hơn?

- Dịch vụ sức khỏe và sức khỏe tâm thần?

- Dịch vụ giáo dục?

- Dịch vụ vận chuyển?

- Dịch vụ nhà ở?

- Dịch vụ việc làm?

2. Làm thế nào để các nhà cung cấp dịch vụ chăm sóc sức khỏe có thể hỗ trợ những người chăm sóc tốt hơn?
